# Supplementary material for: The genome sequence of the Gorse Wanderer, Brachmia blandella (Fabricius, 1798) (Lepidoptera: Gelechiidae)
Source: Wellcome Open Res. 2025 Oct 6;10:551. [Version 1] doi: 10.12688/wellcomeopenres.24971.1 (PMC12963829; doi:10.12688/wellcomeopenres.24971.1)
Supplement: Peer review history

# Reviewer report: Reviewer response for version 1

**DOI:** 10.21956/wellcomeopenres.27506.r147853

**Published:** 2026-03-18

**Authors:** Ricardo Rodriguez-de-la-vega

**Affiliations:** 1 Université Paris-Saclay, Gif-sur-Yvette, France

Comments on Boyes and Boyes "The genome sequence of the Gorse Wanderer..."

 In this Darwin Tree of Life (DToL) project genome note, authors describe the chromosome-level (EBP metric 5.C.Q56*) genome assembly of a *Brachmia blandella *female, a micromoth widely distributed in continental Europe and the British islands. As per usual for DToL's assemblies, this was obtained based on HiFi reads from a single specimen assembled with hifiasm --primary further scaffolded based on Arima-HiC reads (from another specimen) and manual curation. When first published, this was the eighth Gelechiidae genome and the fourth with both Z and W sex chromosomes.

 Overall, the reported assemblies are standard in terms of DToL genome notes. Among the few instances that stand out, I would highlight the following that may warrant further investigation:

 - the smaller than usual contig N50 (below the 1e6 benchmark)

 - the large number of low-coverage contigs that also correspond to the no-hit category in the BUSCO analysis (I suspect these were not placed on the chromosomes)

 - the excess of duplicated BUSCO (2.1% vs ~1% for other published Gelechiidae genome notes)

 - the large number of scaffolds for both the primary and alternate assemblies (900 and 3610, respectively)

 No cobionts were assembled, albeit both mash screen and MarkerScan suggest that contaminants made up to the assembly (see *Brachmia blandella *entry at tolqc.cog.sanger.ac.uk). The alternate haplotype is 5% smaller than the primary assembly, partially explained by the presence of the Z chromosome in the primary assembly only.

 I am including the links to Genomes on a Tree and Tree of Life quality check pages as I think are useful point to access the numerous underlying data analyses.

 (Refer to reference no. 1&2)

 *Read as:

 1e5 bp < contig N50 < 1e6 bp -> 5

 Chromosome N50 <= scaffold N50 -> C

 Consensus quality -> 1 error in ~4e5 bases

Are sufficient details of methods and materials provided to allow replication by others?

Yes

Is the rationale for creating the dataset(s) clearly described?

Yes

Are the datasets clearly presented in a useable and accessible format?

Yes

Are the protocols appropriate and is the work technically sound?

Yes

Reviewer Expertise:

Genomics, Bioinformatics, Evolution

I confirm that I have read this submission and believe that I have an appropriate level of expertise to confirm that it is of an acceptable scientific standard.

## References

[1] : Brachmia blandella – GenomeHubs Record. *Available at: https://goat.genomehubs.org/record?recordId* .–;

[2] : Brachmia blandella – Darwin Tree of Life (genome resource page). *Available at: https://tolqc.cog.sanger.ac.uk/darwin/insects/Brachmia_blandella/* .–;

# Reviewer report: Reviewer response for version 1

**DOI:** 10.21956/wellcomeopenres.27506.r147849

**Published:** 2026-03-05

**Authors:** Wai Lok So

**Affiliations:** 1 The Hang Seng University of Hong Kong (Ringgold ID: 272025), Hong Kong, Hong Kong

The current manuscript presents a genome assembly of the moth, *Brachmia blandella*. The ultra-low-input library preparation and subsequent assembly methods are technically sound. The assembly also includes both Z and W sex chromosomes and a complete mitochondrial genome. The resulting resources would be useful for research on the evolution of Lepidoptera and related genomic studies.

 I have a few comments listed below:

 - Please kindly specify if the sample used for Hi-C is a male or female.

 - Please kindly include a scale bar for the sample in Figure 1, considering this sample is small-sized.

 - Figure 6 shows some "no hit" as small dots in the plot. Can the authors address these contents in the assembly? Are they contaminations or repeats?

Are sufficient details of methods and materials provided to allow replication by others?

Yes

Is the rationale for creating the dataset(s) clearly described?

Yes

Are the datasets clearly presented in a useable and accessible format?

Yes

Are the protocols appropriate and is the work technically sound?

Yes

Reviewer Expertise:

Evolution, genomics, arthropods

I confirm that I have read this submission and believe that I have an appropriate level of expertise to confirm that it is of an acceptable scientific standard.

# Reviewer report: Reviewer response for version 1

**DOI:** 10.21956/wellcomeopenres.27506.r138222

**Published:** 2025-12-11

**Authors:** Will Nash

**Affiliations:** 1 School of Biology, University of East Anglia (Ringgold ID: 6106), Norwich, England, UK

The manuscript describes a chromosome level assembly of a lepidopteran genome. It has been sequenced and assembled using cutting-edge methods. 

 The genome is build from PacBio Hifi data and scaffolded using Hi-C data. There is no anntation. 

 The assembly is 499Mb big. 96% is assigned to 31 Chromosome level scaffolds. The assembly is QV57.5, 98% kmer complete, and 96.9% of the lepidoptera_odb10 BUSCO set at returned. 

 This genome has been sequenced as part of DToL. The methods are technically sound, although it would have been ideal if RNA-seq data could have been generated to annotate the genome. Wet-lab, seqeuncing, and assembly methods are clear and repeatable. Data is all available under PRJEB71264, although I note that a different Common name has been used here, which could be confusing for some readers.

Are sufficient details of methods and materials provided to allow replication by others?

Yes

Is the rationale for creating the dataset(s) clearly described?

Yes

Are the datasets clearly presented in a useable and accessible format?

Yes

Are the protocols appropriate and is the work technically sound?

Yes

Reviewer Expertise:

Evolutionary Genomics, Population Genomics, Genome Assembly, Bioinfromatics

I confirm that I have read this submission and believe that I have an appropriate level of expertise to confirm that it is of an acceptable scientific standard.
